# Anthropometric Characteristics of Spanish Professional Basketball Players

**DOI:** 10.1515/hukin-2015-0038

**Published:** 2015-07-10

**Authors:** Vaquera Alejandro, Santos Santiago, Villa José Gerardo, Morante Juan Carlos, García-Tormo Vicente

**Affiliations:** 1 Departamento de Educación Física y Deportiva. Universidad de León. Spain.; 2 Institute of Sport & Exercise Science. University of Worcester. UK.

**Keywords:** basketball, playing position, body composition, skinfolds, sports level

## Abstract

The study of elite basketball players’ anthropometric characteristics alongside those of body composition contributes significantly to their profiling as professional athletes and plays an important role in the selection process, as these characteristics can have a significant impact on performance. In the current study, 110 professional basketball players from a series of Spanish professional Leagues (ACB, LEB and EBA) and youth level National Teams (U20 and U18) had their anthropometric profiles measured and compared to determine differences between them. Furthermore, all 110 players were divided into three different categories according to their playing position: guards, forwards and centres. The results obtained show no significant differences between players in different competitions in weight, height and the sum of skinfolds. Nonetheless, there were several differences related to body fat content (13.03% in ACB players and 10.52% in the lower categories and National Teams). There were also several differences found between the different playing positions amongst all playing levels in body mass (79.56 ± 2.41, 91.04 ± 1.51 and 104.56 ± 1.73 kg), height (182.28 ± 0.96, 195.65 ± 1.00 and 204.08 ± 0.67 cm), skinfold distribution and perimeters. However, there were no significant differences in body fat content between the different playing positions. The conclusions obtained from this study provide a better understanding to basketball specialists regarding the selection process of players at the elite level, especially on the transition from youth elite programs to men’s elite leagues.

## Introduction

The determination of the anthropometric profile of athletes has become one of the fundamental focuses within different studies in the field (Dezman et al., 2001; Ostojic et al., 2006; Montgomery et al., 2008; Vuckovic and Mekic, 2009). The anthropometric profile of basketball players has been shown to be a key determining factor in the selection process and not just as a predictor of performance (Hoare, 2000; Bayios et al., 2006). Within this process, Dezman et al. (2001) stated that height was the variable that traditionally determined the specific position of each of the players. A players’ position on the court is highly determined by their size and height. Traditionally the tallest players will play the role of a centre or forward, close to the basket, whereas those of a shorter stature will play on the perimeter, further away from the basket (Sallet et al., 2005; Ostojic et al., 2006).

Many studies have linked the anthropometric profile and body composition to the physical and physiological parameters shown by players (Sallet et al., 2005; Drinkwater et al., 2008). Furthermore, Ostonjic (2002) demonstrated that players with lower body fat content would invariably perform at a higher level than those with higher body fat. Additionally, basketball players who were taller in their stature, heavier and had longer limbs performed at a higher athletic standard (Sisodiya and Yadaf, 2010). Moreover, body mass and height of elite basketball players are not the only parameters that literature has focused on. Other variables such as body perimeters and diameters have been taken into account when examining the anthropology of elite basketball players. Accordingly, Dizdar et al. (1995) found in their research that players playing in the centre position showed higher values in body perimeter and diameter than those playing in the guard and forward positions. This aspect was further backed by Mekic and Vukovic (2009) as they confirmed these findings, showing significant differences in all perimeters (thigh, calf, arm and forearm circumferences) between guards, forwards and centres.

The evolution of rules and technical-tactical fundamentals in basketball has resulted in greater physiological and anthropometric differentiation between particular playing positions, thereby making it essential to study the players as a function of their different playing positions (Sallet et al., 2005; Ben Abdelkrim et al., 2007; Rodriguez et al., 2007; Cormery et al., 2008; Vaquera et al., 2008). Therefore, the aim of the current study was to compare the anthropometric profile and body composition of Spanish elite basketball players from different categories and different playing positions. Furthermore, a secondary aim of this study was to aid the player selection process, in which basketball specialists look for transition players, playing in all three positions, from youth elite programs to the professional men’s level.

## Material and Methods

110 Spanish professional basketball players from different elite professional leagues and categories volunteered to participate in this study. Their respective teams were previously selected to be a part of the current study and all participants completed a health-screening questionnaire and provided fully informed consent prior to the commencement of the study. The current research was conducted during the competitive microcycle of the regular season (ACB league, LEB league and EBA league) and during the final phase of preparation before the start of the European Championships (U18 and U20 Spanish National squads). Each referee was informed about the research design and the requirements, benefits and risks of the study. All procedures were conducted in accordance with approval of the Human Ethics committee of the Leon University, according to the declaration of Helsinki.

The procedures used in order to obtain the anthropometric variables determined in the current study followed the GREK-ISAK methodology (Spanish Group of Anthropometry and International Society for the Advancement of Kinanthropometry). The variables described here are as follows: age (years), body mass (kg; a COBOS Precision Scale, model 50K150), body height (cm; Stadiometer Detecto, model D52, USA), and skinfolds (mm Harpenter® Adipometer, British Indicators LTD, UK). Flexed and contracted arm perimeters were measured along with these of the thigh and calf. The measurements were conducted using a Holtain British Indicators LTD (UK) metric tape. Whereas the diameters (cm) measured were those of the Biesoloideo, and the Biepicondylar of both the humerus and the femur. The summations of the triceps, subscapular, iliac crest, abdominal, thigh and calf folds were obtained. In addition all 8 folds were added (the previous 6 in addition to the axilla and biceps). Body fat percentage was estimated according to the equations described and validated by the ISAK-GREK methodology (Juhasz, 1974): % Body Fat (male) = 3.64 + (Σ6 skinfolds (mm) × 0.097)). All measurements were taken at 09:00 am, after an overnight fast.

The graphic data and statistic processing were implemented in Excel spreadsheet V 7.0 and SPSS V 15.0, respectively. The results are shown as mean values ± standard error of the mean (S.E.M.). The studied parameters were all analysed using an ANOVA-MANOVA test, wherein the values for p < 0.05 were considered as significant.

## Results

Table 1 shows the results obtained in relation to age, body mass, height and BMI (body mass index) of the players from different leagues. ACB players were found to be heavier than those playing in LEB (1%) and between 3% and 8% heavier than both Junior National squads evaluated in the current study as well as EBA players. ACB players ranked lower in regard to height, while LEB and National squad players were slightly taller (1.6% and 0.6%, respectively). This finding is somewhat surprising because generally players in the top divisions are taller than those in the lower divisions (Cormery et al., 2008). EBA players recorded the shortest statures in this study. In reference to the BMI, ACB players had the highest values in relation to other leagues, the difference being 4.5% (National squads) and 2.1% (LEB).

Data with regard to body height, mass and the BMI based on the player specific position are shown in Table 2. Results indicate that 12.7% of the Guards were significantly lighter than the forwards and 24.2% lighter than the Centres (p < 0.05). Likewise the Forwards were 12.9% (p < 0.05) lighter than the Centres. With respect to the height of the players, the Guards were 6.6% shorter than the Forwards and 11% shorter than the Centres. Moreover, the Forwards were 4.2% (p ≤ 0.03) shorter than the Centres. However, when analysing the BMI, it is important to note that there were no significant differences between player positions within the different leagues. Nevertheless the Centres showed higher values (5%) than the Guards and Forwards.

**Figure 1:**
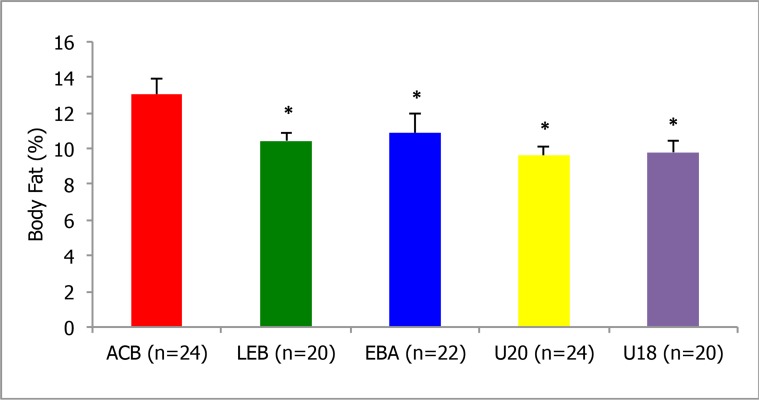
Body fat % obtained in the anthropometric analysis of the different categories of the Spanish basketball leagues and National squad programs and average values of the 110 professional players. Significant differences: ^*^ = p <0.05 with respect to the ACB League.

Figure 1 shows players’ percentage of body fat in all categories and levels used in this study. Significant differences (p < 0.05) were found between the percentage of body fat in ACB players (13%) and the remaining categories (10.5%). Whereas no significant differences were observed when measuring body fat in all three playing positions (around 10%)

Analysing the summation of 6 and 8 skinfolds (Σ of 6 and 8 folds) in each of the five categories of professional basketball players in Spain, no significant differences were found in any of them, with values between 60 and 80 mm in Σ of 6 folds, with a tendency to be lower in younger players (U18 and U20 Spanish National squads). No significant differences were found in the sum of 6 and 8 skinfolds between different playing positions, yet they increased from the Guard to the Centre between 7 mm (7–11%) in the 6-fold Σ, and 4–10 mm (5–11%) in Σ 8 skinfolds.

**Figure 2:**
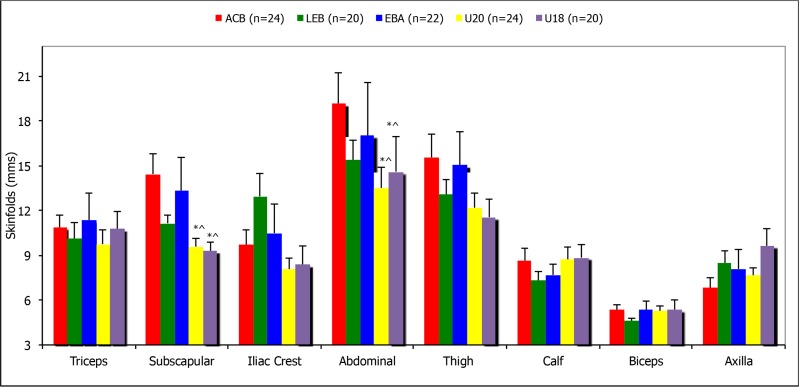
Skinfolds in the different Spanish basketball categories. Mean values ± S.E.M. Significant differences: ^*^ = p <0.05 with respect to the ACB League. Significant differences: ^ = p <0.05 compared to the EBA League.

Skinfolds of particular categories analysed (Figure 2) demonstrate the existence of significant differences only in the subscapular measurement with ACB and EBA players reaching 13–14% in contrast to the 9% obtained by the U18 National squad (p < 0.05). Furthermore, a significant difference (p < 0.05) in abdominal fold was obtained between ACB and EBA players (17–19%) and the U18 national squad (13–14%). However, the lack of statistical significance demonstrates the homogeneity of the skinfold measurements regardless of the competitive level.

It is worth pointing out that the variability in skinfolds between playing levels was small. There were considerable differences when measuring the skinfolds of different playing positions (26% in abdominal skinfolds, 28% in suprailiac and 20% in the triceps skinfold). The highest fat content was found among the Centres.

Table 3 shows the 3 perimeters and 3 diameters of professional basketball players at different levels evaluated in the current study. There were no significant differences in the perimeters while significant differences were obtained in all diameters which were smaller in the Spanish National squad (U18) than in other categories (p < 0.05).

Additionally, the perimeters measured indicated that the Centres had significantly higher values than those of the Forwards and Guards (Figure 3). The upper arm perimeter in the Centres was 6% higher than in the Guards and 5% higher than in the Forwards. Thigh circumference was found to be 7% greater in the Centres in comparison to the Guards, showing no difference between the Centres and the Forwards.

## Discussion

The current study was conducted in Spain, where some of the best basketball competitions in all of Europe reside, attracting elite players from all over the globe. As a result the authors hoped to obtain concluding figures in anthropometrical measurements of players and to determine how these impact the game. Additionally, the Spanish Junior elite programmes have obtained the best results in European and World championships in the last decade (FIBA, 2014). Therefore, a further investigation into these junior athletes was undertaken in order to establish key figures in the player selection process as well as that of the transition period in junior to senior elite programs.

One of the main findings of this study is that of the body fat content found in players in the top Spanish division (ACB), which was significantly higher than their counterparts in this other leagues analysed. When comparing body fat percentage with players in LEB and EBA as well as U20’s and U18’s the current study found there to be significant differences, a fact that is quite surprising given that these leagues present lower levels of competition when compared to the ACB. It is also a notable finding, when compared to previous studies carried out in other European leagues; it was found that top players also had a higher body fat content, although their values were higher than in ACB players (Vuckovic and Mekic, 2009; Cormery et al., 2008; Ostojic et al., 2006). It could be suggested that upon reaching the top leagues, players use their mass to maintain their position on the court, on defence and offence (Gerodimos et al., 2005; Drinkwater et al., 2008). Moreover, what is noteworthy about this finding is that when comparing the younger playing categories of U20’s and U18’s to the English U16 national squad there is almost a 2% difference in body fat content (around 10.5% in Spain’s national squads to the 12.6% in their English counterparts) (Delextrat and Cohen, 2008). Given the success that the Spanish junior national squad had in the last decade in international basketball, this could be one of the main factors contributing to that success at the junior and senior levels alike. The differences in body fat found between senior players (ACB, LEB and EBA) and young players (U20 and U18) can be attributed to the natural increases in body fat with age (McArdle et al., 1991).

The BMI has been found to be very similar across top leagues in Europe (Vuckovic and Mekic, 2009; Cormery et al., 2008; Ostojic et al., 2006; Sallet et al., 2005) and it showed to be important at the elite level and in the player selection process. The current study demonstrates that ACB and LEB players had higher values of the BMI than the rest of players analysed. Moreover, players from the Junior Elite programmes had noticeably lower BMI values than the rest of the players tested. This finding had a twofold interpretation i) they hadn’t developed and grown fully into their adult bodies and ii) in order for them to make a top elite division they had to increase their BMI preferably adding muscle mass.

Additionally, when analysing different playing positions we found that the BMI was very similar across all leagues between the guards and the forwards; however, the centres demonstrated higher levels of the BMI. The role of a Center is really important within the basketball court as they are the cornerstones both offensively and defensively, and one will find that in most successful basketball programs, teams have very skilled Centers (George et al., 2009; Sampaio et al., 2006). Compared to other European leagues, the ACB and LEB are better structured and teams benefit from higher financial support which can provide better coaching and assisting staff, as well as more advanced technology. Moreover, if teams were to look closer at body mass of their Centers while still maintaining their muscle mass, it could lead to better performance on the court. To further assist this point of view, results from the summation of the skinfolds should also be considered.

Skinfold analysis in basketball provides useful information. However, it is important not to rely on equations or formulas derived from general populations. In the present study, skinfold analysis was carried out, and although some consideration was given to the results obtained from the summation of skinfolds, it was their distribution that was of our main interest. The sums of 6 and 8 skinfolds show no significant differences between playing levels and playing positions (sum of 6 skinfolds ranges between 61.84 ± 5.04 and 81.25 ± 6.2 mm whereas the sum of the 8 skinfolds ranges between 74.78 ± 5.82 and 90.89 ± 6.34 mm). When analysing distribution of these skinfolds more differences were found between playing levels and playing positions. More specifically the abdominal, suprailiac and tricep skinfolds presented the biggest differences between playing levels and positions, the position most affected was the Center. Delextrat and Cohen (2008) argued that athletic performance could be diminished significantly by an excess of abdominal fat, and if Centres in top teams managed to shred excess fat, their performance, and more substantially the teams outcomes would improve.

The role of the Center is usually assigned to the tallest players on the team, and height is one of the most commonly associated traits with basketball, but it does not just apply to Centers. Both Guards and Forwards were found to have similar height characteristics across all leagues examined, except in the EBA league, which presents itself as the lowest level league analysed in the current study, were all players were significantly shorter than their counterparts in higher divisions. The findings of this study further reinforce this statement, but also add a new value to it as both Junior teams showed slightly higher profiles in height than in all three professional leagues. Which also reiterates body height as a valuable indicator in player selection, but not the only one.

## Conclusions and Future Research

The results of this study conclude that there are some anthropological and body composition differences that can significantly improve one’s performance and allow transition to the highest level. With these results, the authors would like to facilitate the player selection process, especially when transitioning from elite youth programs to the professional level. Body fat, skinfold measurement, body height and diameters were found to be key components in the makeup of an elite player and often these were indicators of the level of play and the leagues that each player competed in. Playing positions were determined by anthropological measurements in body mass, height, and body perimeters and were also a significant indicator of the level of play.

Since the year 2000 (last significant change in the FIBA rules) basketball has evolved into a faster and more dynamic game, which has had significant repercussions on the players’ anthropological profiles. The differences have been so significant that the 5 traditional positions can now be placed into 3 positions, as has been done in the current study. The authors envision that as the game faces another natural transition and coupled with new rule changes that will be applied in the near future. However, further analysis of the 5 traditional positions is necessary to acquire more valuable information in terms of player selection and scouting processes.

## Figures and Tables

**Table 1:** Age, body mass, height and BMI in the 110 Spanish professional basketball players analysed in this study. Mean values ± S.E.M.

	ACB League	LEB League	EBA League	Spain NT U20	Spain NT U18

n=110	n=24	n=20	n=22	n=24	n=20
Age (years)	28±1.16	29±0.99	20±0.78	19±0.10	18±0.13
Body mass (kg)	97.95±3.52	96.48±2.37	89.73±2.86	93.44±3.02	94.05±4.12
Height (cm)	195.30±2.69	198.25±2.05	193.50±2.04	196.83±1.93	198.92±1.99
BMI (kg/m^2^)	25.11±1.62	24.59±1.52	24.11±1.21	24.32±1.13	23.97±1.09

**Table 2:** Body mass, height and BMI in the 110 Spanish professional basketball players analysed in this study as function of their playing position. Mean values ± S.E.M. Significant differences: # = p <0.05 between the Guards and Forwards with the Centers and ^*^ = p <0.05 between the Guards with the Forwards

	Guards	Forwards	Centers

n=110	n=21	n=48	n=41
Body mass (kg)	79.56±2.41#^*^	91.04±1.51#	104.56±1.73
Height (cm)	182.28±0.96#^*^	195.65±1.00#	204.08±0.67
BMI (kg/m^2^)	23.98±3.03	23.88±2.88	25.02±2.09

**Table 3:** Perimeters and diameters of the players from the different categories analysed in this study. Mean values ± S.E.M. (n = 110). Significant differences: # = p <0.05 with respect to the Spanish National squad U18.

	ACB League	LEB League	EBA League	Spain NT U20	Spain NT U18

Wrist Diameter (cm)	6.14±0.1#	6.14±0.11#	6.11±0.1#	6.03±0.06#	5.5±0.09
Humeral Diameter (cm)	7.45±0.1#	7.51±0.1#	7.19±0.73#	7.28±0.07#	6.74±0.09
Femoral Diameter (cm)	10.46±0.2+	10.44±0.14#	10.38±0.09#	10.23±0.04#	10.05±0.14
Arm Perimeter (cm)	37.52±0.56	35.75±0.42	33.64±0.86	35.35±0.44	33.46±0.52
Thigh Perimeter (cm)	61.46±1.21	58.92±0.71	56.6±2.32	58±0.86	57.3±1.4
Leg Perimeter (cm)	40.1±0.56	39.21±0.62	39.87±0.6	39.58±0.58	40.21±0.62
